# Supplementary material for: Empirical methods for controlling false positives and estimating confidence in ChIP-Seq peaks
Source: BMC Bioinformatics. 2008 Dec 5;9:523. doi: 10.1186/1471-2105-9-523 (PMC2628906; doi:10.1186/1471-2105-9-523)
Supplement: Additional File 1 — A variety of html documents from the USeq web site detailing the available applications, their best usage, output file type descriptions, command line menus, etc. [file 1471-2105-9-523-S1.zip › USeqUserGuides/JavaDoc.zip!/JavaDoc/edu/utah/seq/data/package-summary.html]

edu.utah.seq.data


|  |  |  |
| --- | --- | --- |
| **Overview** \| **Package** \| Class \| **Use** \| **Tree** \| **Deprecated** \| **Index** \| **Help** | |  |
| **PREV PACKAGE**   **NEXT PACKAGE** | **FRAMES**    **NO FRAMES**     **All Classes** |  |


---

## Package edu.utah.seq.data

Helper classes for working with binary PointData, EnrichedRegions, etc.

**See:**
  
          **Description**

| **Class Summary** | |
| --- | --- |
| **ComparatorEnrichedRegionPosition** |  |
| **ComparatorEnrichedRegionScore** |  |
| **ComparatorInfoPoint** | Class to sort Info by chromosome. |
| **ComparatorPointPosition** | Class to help sort an array of Window[] by chromosome, start position, length shortest first. |
| **ComparatorSmoothingWindowPosition** |  |
| **ComparatorSmoothingWindowScore** |  |
| **ComparatorSmoothingWindowTwoScore** | Sorts by scores[0], then scores[1] |
| **DividePointData** | Merges Point Data and then divides using random sampling. |
| **EnrichedRegion** |  |
| **FilterPointData** | Filters PointData for those that intersect a list of regions, e.g. |
| **HeatMapMaker** | Converts a SmoothingWindow[] into a heat map specific bar file for import into IGB. |
| **HeatMapMakerPosNeg** | Converts a SmoothingWindow[] into a heat map specific bar file for import into IGB. |
| **Info** | Data file information. |
| **InfoPoint** |  |
| **Point** |  |
| **PointData** | Container for holding genomic positions - scores data. |
| **Simulator** |  |
| **SmoothingWindow** | Coordinates are interbase! |
| **SmoothingWindowInfo** | Chromosome specific container for window level results from EnrichedRegionMaker. |
| **SubSamplePointData** |  |

## Package edu.utah.seq.data Description

Helper classes for working with binary PointData, EnrichedRegions, etc.

---


|  |  |  |
| --- | --- | --- |
| **Overview** \| **Package** \| Class \| **Use** \| **Tree** \| **Deprecated** \| **Index** \| **Help** | |  |
| **PREV PACKAGE**   **NEXT PACKAGE** | **FRAMES**    **NO FRAMES**     **All Classes** |  |


---
